# Comprehensive geriatric assessment is associated with increased antidepressant treatment in frail older people with unplanned hospital admissions—results from the randomised controlled study CGA-Swed

**DOI:** 10.1186/s12877-022-03324-9

**Published:** 2022-08-05

**Authors:** Theresa Westgård, Isabelle Andersson Hammar, Katarina Wilhelmson, Margda Waern

**Affiliations:** 1 Department of Psychiatry and Neurochemistry, Institute of Neuroscience and Physiology, The Sahlgrenska Academy, University of Gothenburg, Gothenburg, Sweden; 2 Department of Occupational Therapy and Physiotherapy, Sahlgrenska University Hospital, Göteborgsvägen 31, SE-431 80, Mölndal, Sweden; 3 Centre of Aging and Health-AGECAP, University of Gothenburg, Gothenburg, Sweden; 4 Department of Health and Rehabilitation, Institute of Neuroscience and Physiology, The Sahlgrenska Academy, University of Gothenburg, Box 455, 405 30 Gothenburg, Sweden; 5 The Geriatric Development Centre, The Sahlgrenska University Hospital, Region Västra Götaland, 413 45 Gothenburg, Sweden; 6 Department of Acute Medicine and Geriatrics, The Sahlgrenska University Hospital, Region Västra Götaland, 413 45 Gothenburg, Sweden; 7 Psychosis Clinic, Region Västra Götaland, Sahlgrenska University Hospital, Gothenburg, Sweden

**Keywords:** Mental health, Depressive symptoms, Montgomery-Åsberg Depression Rating Scale, Frail older people

## Abstract

**Background:**

Frail older people are at higher risk of further deterioration if their needs are not acknowledged when they are acutely ill and admitted to hospital. Mental health comprises one area of needs assessment.

**Aims:**

The aims of this study were threefold: to investigate the prevalence of depression in frail hospital patients, to identify factors associated with depression, and to compare depression management in patients receiving and not receiving Comprehensive Geriatric Assessment (CGA).

**Methods:**

This secondary analysis from the CGA-Swed randomized control trial included 155 frail older people aged 75 years and above**.** Instruments included Montgomery Åsberg Depression Rating Scale (MADRS), the ICE Capability measure for older people **(**ICECAP-O) and the Fugl-Meyer Life Satisfaction scale (Fugl-Meyer Lisat). Depression was broadly defined as MADRS score ≥ 7. Regression models were used to identify variables associated with depression and to compare groups with and without the CGA intervention.

**Results:**

The prevalence of a MADRS score indicating depression at baseline was 60.7%. The inability to do things that make one feel valued (ICECAP-O) was associated with a fourfold increase in depression (OR 4.37, CI 1.50–12.75, *p* = 0.007). There was a two-fold increase in odds of receiving antidepressant medication in the CGA intervention group (OR 2.33, CI 1.15–4.71, *p* = 0.019) compared to patients in the control group who received regular medical care.

**Conclusion:**

Symptoms of depression were common among frail older people with unplanned hospital admission. Being unable to do things that make one feel valued was associated with depression. People who received CGA intervention had higher odds of receiving antidepressant treatment, suggesting that CGA improves recognition of mental health needs during unplanned hospital admissions in frail older people.

**Trial registration:**

ClinicalTrials.gov, NCT02773914. Retrospectively registered 16 May 2016.

## Background

The needs of frail older people are multifaceted, and may be missed by the health care system [1] which is mainly designed to address one medical issue at a time [2]. Frailty is an age-related decline across multiple physiological systems, which results in a state of decreased reserve resistance to stressors [3, 4]. Frail older people have a higher likelihood of clinical depression and anxiety compared to older people who are not frail [5]. Depression is associated with adverse health outcomes [6]. However, diagnosing depressive syndromes in frail older persons can present a challenge for the clinician [7]. All too often symptoms of depression and anxiety are missed in these patients, despite being common in occurrence [6, 8]. When frail older people’s needs are not addressed they are at higher risk of further deteriorating health [9]. Therefore, assessment of needs should include evaluation and treatment of frail older people’s mental health issues [6].

Clinicians have been encouraged to increase their focus and improve detection of depression and anxiety symptoms in older people [6], especially frail older people who are at particular risk and would benefit from such detection when admitted to hospital for unplanned care. Depression seems common in older physically ill patients in general hospitals, but risk estimates vary widely (5–58%) [10]. Depression has been found to unfavourably affect the outcome of a numerous medical conditions, to reduce treatment compliance, to impair rehabilitation, and to decrease survival [10]. However, the optimal approach to the treatment of depression in frail older adults remains to be elucidated. The authors of a recent systematic review [11] concluded that the efficacy of antidepressants in adults aged 65 and older is questionable. While the meta-analysis did not involve persons with frailty, results could have clinical implications for frail older persons, who might be even more vulnerable to side effects than their non-frail peers.

The relationship between frailty and depression is convoluted; the two constructs are distinct but also overlap [7]. This is because depression as a syndrome has medical signs and symptoms that can be interrelated and may be difficult to clinically separate from frailty in advanced old age. Shared symptoms include fatigue, weight loss, and decreased engagement in activities of daily living (ADL), which could be related to decreased energy reserve or loss of ability to engage in ADL [7]. In a recent meta-analysis [5], the overall prevalence of depression with frailty was nearly 39%, and a similar proportion of people with depression were also frail. Studies of the co-occurrence of frailty and depression in hospital settings are uncommon but warranted [5]. We employed data from a Comprehensive Geriatric Assessment (CGA) randomized control trial (RCT), the CGA-Swed [12], to explore depression symptoms in frail older adults with unplanned hospital admissions. CGA is designed to assess older people’s medical, psychological, social, and functional capabilities and identify the challenges they face through the coordination of multidimensional diagnostics by an interdisciplinary treatment team [13–15]. To date, CGA remains the most effective intervention for frail older people [16, 17] and is recommended as a person-centered approach to meet the complex care needs of older adults with comorbidities and dependence in ADL [18, 19]. CGA is a systematic evaluation of frail older people by a team of health professionals whose purpose is to gather data, have team discussions, develop a treatment plan, implement the treatment plan, while monitoring and making revisions to the plan as needed [20]. CGA has been largely studied in hospital, home care, and clinic settings. While virtually all trials of this model have resulted in improved detection and documentation of problems in the frail older population, mental health outcomes are sparsely studied in the context of CGA [20]. We identified one hospital-based study involving a mixed sample of robust and frail older people that reported frail older people had 5 times higher odds (OR 5.07) of having depressive symptoms than the older patients who were not frail [21].

Moreover geriatric assessments have been found to be inconsistent or as having limited-quality patient-oriented evidence when screening older people for depression, despite having access to the appropriate support measures [20]. While CGA does not screen for depression per se, it could be useful in identifying depression, since it is comprehensive. The aims of this study were threefold: to investigate the prevalence of depression (broadly defined as a MADRS score ≥ 7) in frail hospital patients, to identify factors associated with depression, and to compare depression management in patients receiving and not receiving Comprehensive Geriatric Assessment (CGA).

## Methods

### Study design

The study is based on data from a two-armed RCT, (the CGA-Swed described in the study protocol [12]) involving frail older people with unplanned admissions at a Swedish university hospital. Participants were randomized to the CGA intervention or the control group receiving routine medical care. All data was collected from March 2016 until January 2020. Ethics approval was obtained, ref. no: 4 899–15, Regional Ethical Review Board in Gothenburg, Sweden. Trial Registration: Clin.Trial.gov NCT02773914.

### Participants and setting

Eligible for inclusion were frail older people aged 75 or older who sought emergency department care at a Swedish university hospital and required an unplanned medical hospital admission. Exclusion criteria were admission via fast track (stroke, coronary infarct, or hip fracture). For more information and details about the study, see the pilot and study protocol [12, 22].

### Recruitment, consent and randomization

Eligible participants were identified by using the FRESH screening tool [23]. Potential participants were invited to join the study by the care coordinator in the emergency department. Information was provided both verbally and in writing. Those who agreed to participate signed a consent form. Some of the participants had cognitive impairments that made them unable to understand the information well enough to give their consent. This impairment was usually determined by the care coordinator during the inclusion. In these cases, their next of kin signed the consent form.

Randomization was allocated by computer-generated number which then was assigned by the care coordinator. Allocation was concealed by using opaque sealed envelopes that were numbered sequentially. The researchers, (trained as occupational therapists, physiotherapists, nurses and physicians, working with in academia) performed the interviews and measurements during hospital stay All were trained in observing and assessing in accordance with the guidelines for frailty and the outcome measurements.

### Sample

In all, 178 people aged 75 years or older who required an acute medical hospital admission were randomized in the CGA-Swed RCT [12]. In total, 155 people (65 men and 90 women) were eligible and included into the study, with an allocation ratio of 1:1, resulting in 77 in the control group and 78 in the CGA intervention group.

#### Procedures

The intervention was CGA performed on a geriatric ward by a multidisciplinary team and included comprehensive assessments a person’s of medical, functional, psychological, social, and environmental status as well as treatment, rehabilitation, discharge planning, and follow-up. The team consisted of a geriatrician, a registered nurse, an assistant nurse, a physiotherapist, and an occupational therapist, and when needed included a social worker and a dietician. The multidisciplinary teamwork was person-centred. A team meeting was held every weekday, where the team shared information and used their experience and competence to tailor the care to the needs of each frail older patient. During the discharge planning, the CGA-ward could recommend follow-ups to be carried out by primary care and/or social care providers [19].

The control group received ordinary medical care on a ward at the same hospital. On this ward, there was no geriatrician and the staff did not work according to CGA and did not use a specialised multi-disciplinary team. The control ward had access to a physiotherapist and occupational therapist on a when-in-need basis.[19].

### Assessment of depressive symptoms

The Montgomery-Åsberg Depression Rating Scale (MADRS) [24] was used to rate symptoms of depression. Participants responded verbally to the questions asked by the researchers. Items (1. Apparent sadness 2. Reported sadness 3. Inner tension 4. Reduced sleep 5. Reduced appetite 6. Concentration difficulties 7. Lethargy 8. Inability to feel 9. Pessimistic thoughts and 10. Suicidal thoughts) are rated 1–6. MADRS has good test–retest agreement; the reliability of all ten items was good to excellent and reliability for the total MADRS score was excellent [25].

### Associated factors

Factors potentially associated with depression included activities of daily living (ADL), self-rated health (SRH), two questions specific to attachment and roles, two questions specific to satisfaction with physical and mental health, and the somatic organ category scores excluding psychiatric illness score using the Cumulative Illness Rating Scale-Geriatrics (CIRS-G) score.

### Activities of Daily Living

Activities of Daily Living (ADL) was measured using the ADL staircase [26], which measures five personal ADL (P-ADL) items (bathing, dressing, going to the toilet, transferring and feeding) and four instrumental (I-ADL) items (shopping, cooking, cleaning and transportation).The ADL staircase [27] then scores the participant’s independently managed activities. For the purpose of this study, ADL was scored as dependent, partly dependent or independent, and total score was then dichotomized. Dependent and partly dependent in ADL was coded as dependent and independent ADL was coded as independent.

The ADL staircase has been reported to have good reliability and validity in assessing the functional status, different levels of ability/disability [28], and independence/dependence when performing ADL tasks [26].

### Self-rated health

Self-rated health (SRH) [29] was measured by asking the participants to evaluate their health status based on a single question taken from the SF-36 “In general would you say your health is?: excellent, very good, good, fair or poor”. This question is subjective in nature, and for the purpose of this study, the responses were dichotomized. Excellent, very good and good were coded as a positive response and fair and poor were coded a negative response.

### ICE Capability measure for older people

Two questions addressing attachment (love and friendship) and roles were selected from the ICECAP-O [30], since they measure capability in older people, and focuses on wellbeing defined broadly rather than focusing merely on health [31]. Four alternative statements were available for the participant to choose which best suited their current state related to attachment and role. The attachment options were: I can have all of the love and friendship that I want, I can have a lot of the love and friendship that I want, I can have a little of the love and friendship that I want, I cannot have any of the love and friendship that I want. The role options were: I am able to do all of the things that make me feel valued, I am able to do many of the things that make me feel valued, I am able to do a few of the things that make me feel valued, I am unable to do any of the things that make me feel valued. The responses were then dichotomized agree / disagree. Participants who responded " I can have all of the love and friendship that I want" or "I can have a lot of the love and friendship that I want" were considered to agree. Participants who responded “I am able to do all of the things that make me feel valued” or “I am able to do many of the things that make me feel valued” were considered to agree.

### Satisfaction with physical and mental health

Two questions exploring how a person subjectively experiences their physical and mental health status were used from the Fugl-Meyer happiness and domain specific life satisfaction questionnaire [32]. Participants received a scale with the following responses: very unsatisfied, unsatisfied, a bit unsatisfied, somewhat satisfied, satisfied, very satisfied related to the two questions asked. The responses were then dichotomized (agree / disagree). Participants who responded “somewhat satisfied, satisfied or very satisfied” were considered to agree.

### Mini-mental state examination

The Mini-Mental State Examination (MMSE) [33], is a practical method for rating global cognition. MMSE demonstrates moderately high levels of reliability, and has been reported to be internally consistent [34]. For the purpose of this study participants were considered frail on the basis of cognition if their score was below 25 out of 30 [35].

### Cumulative illness rating scale-geriatrics

Morbidity/disability was measured with the Cumulative Illness Rating Scale-Geriatrics [36] (CIRS-G), a quantitative rating instrument of the chronic medical illness burden modified for geriatric assessments. The CIRS-G includes the 14 following categories: heart, vascular, hematopoietic, respiratory, eyes–ears–nose throat and larynx, upper gastrointestinal, lower gastrointestinal, liver, renal, genitourinary, musculoskeletal, neurological, endocrine, and psychiatric illness. Each organ system was scored as follows: 0. No problem affecting that system, 1. Current mild problem or past significant problem, 2. Moderate disability or morbidity and/or requires first-line therapy, 3. Severe problem and/or constant and significant disability and/or hard-to-control chronic problems or 4. Extremely severe problem and/or immediate treatment required and/or organ failure and/or severe functional impairment. CIRS-G items were assessed by one of the authors (KW) based on data from the research interviews as well as medical record review after discharge. For the purpose of this study, the CIRS-G score were used 1) for the number of ratings scoring 3 or higher excluding psychiatric illness, and 2) as a psychiatric illness score excluding all other organ somatic scores.

### Statistical analysis

In this secondary data analysis, descriptive and analytical statistics were used to compare the means between men and women and the CGA intervention group with the control group. Two-sided tests were used, and a value of p ≤ 0.05 was considered statistically significant. Univariate analyses were performed to investigate the association between depression (MADRS ≥ 7) and gender, ADL total score, SRH, CIRS-G total score (without psychiatric illness), ICECAP-O (two questions) and Fugl-Meyer (two questions) with Mann–Whitney U tests, since the data was not normally distributed. Only those variables that showed a significant relationship with depression were subsequently tested in the final logistic regression model (enter). All statistical analyses were performed using SPSS for Windows software package, version 22.0 (SPSS Inc., Chicago, IL, USA).

## Results

The participants had a mean age of 86, ranging from 75–101 years. The proportion of women (58%) was somewhat larger than men. Women were more likely to live alone (Table 1). All of the men and 90% of the women required assistance with activities of daily living. Men reported better self-rated health compared to women. Half of the participants had impaired cognition (MMSE score < 25). The latter was more common in men, affecting almost two-thirds.

**Table 1:** Characteristics of participants, by gender

Baseline Characteristics	Men (*n* = 65)		Women (*n* = 90)		*p*-value
**Age, mean (range)**	85.1	(76–100)	87.2	(75–101)	
**MMSE**^**a**^**, mean (range)**	23.6	(13–30)	24.6	(9–30)	
	**N**	**(%)**	**n**	**(%)**	
**Living alone**	29	(44.6)	70	(77.8)	**0.000**
**Tertiary education**	11	(16.7)	10	(11.1)	0.300
**Independent in ADL**	0	(0)	9	(10.0)	-
**Good self-rated health **^**b,c**^	26	(40)	21	(23.9)	**0.027**
**Pre-frail**^**d**^	4	(6.2)	5	(5.6)	0.875
**Frail**^**d**^	61	(93.9)	85	(94.4)	0.875
**Frailty indicators**^**d**^					
** Weakness**^**c**^	23	(35.9)	27	(30.7)	0.540
** Fatigue**	54	(83.1)	84	(93.3)	0.051
** Weight loss**^**c**^	33	(50.8)	45	(50.6)	0.980
** Reduced physical activity**^**c**^	45	(75.0)	60	(69.0)	0.972
** Impaired balance**^**c**^	58	(90.6)	81	(91.0)	0.897
** Reduced gait speed**^**c**^	48	(75.0)	75	(83.3)	0.206
** Impaired vision**^**c**^	51	(82.3)	68	(77.3)	0.722
** Impaired cognition**^**c**^	41	(64.1)	35	(39.8)	**0.003**

^a^(MMSE) Mini-mental state examination

^b^Self-rated health (excellent/very good/good)

^c^Missing: self-rated health 2 women; weakness 2 women, weight loss one missing from women, reduced physical activity 3 women, impaired balance 1 man, 1 woman, reduced gait speed 1man, impaired vision 3 men, 2 women and impaired cognition 1 man, 2 women

^d^Frailty indicators measured weakness, fatigue, unintentional weight loss, reduced physical activity, impaired balance, reduced gait speed, visual impairment, and impaired cognition, and were categorized as non-frail (0 indicators), pre-frail (1–2 indicators), and frail (≥ 3 indicators). Cut-off levels of frailty indicators were *Weakness*: Reduced grip strength considered to be below the lowest norm range for ages 80–84, 13 kg for women and 21 kg for men for the right hand, and below 10 kg for women and 18 kg for men for the left hand, using a North Coast dynamometer [37]. *Fatigue*: Question from the Göteborg Quality of Life Instrument [38], answering “Yes” to the question “Have you suffered any general fatigue/tiredness over the last three months?”. *Weight loss*: Question from the Göteborg Quality of Life Instrument [38], answering “Yes” to the question “Have you suffered any weight loss over the last three months?”. *Reduced physical activity*: Taking 1–2 or fewer outdoor walks per week. *Impaired balance*: The Berg Balance Scale [39], reduced balance defined as having a value ≤ 47. *Reduced gait speed*: Walking four meters with a gait speed ≤ 0.6 m/second [40]. Vision was measured with the KM chart [41], impaired vision defined as having a visual acuity of 0.5 or less. Impaired cognition defined as a score below 25 on the Mini-mental state examination (MMSE) [33, 35]

Of the 135 participants assessed with MADRS, the proportion with MADRS ≥ 7 was 60.7%, (males 66% and female 57%). Most had symptom burdens corresponding to mild depression (Table 2). Men had more severe scores on the CIRS-G psychiatric category and reported greater loss of pleasure in life. Poor appetite was more common in women.

**Table 2:** Symptoms of morbidity/disability in frail older men and women with acute hospital admissions

	**Men (*n* = 65)** **n (%)**		**Women (*n* = 90)** **n (%)**		***p*-value**
MADRS^a,b^ Normal	19	(34.5)	34	(42.5)	0.353
MADRS Mild depression	33	(60)	37	(46.3)	0.117
MADRS Moderate depression	3	(5.5)	9	(11.3)	0.255
MADRS Severe depression	0	-	0	-	-
CIRS-G^c^ Psychiatric illness no problem	8	(12.3)	17	(18.9)	0.275
CIRS–G Psychiatric illness current or previous problem	11	(16.9)	19	(21.1)	0.516
CIRS-G Psychiatric illness moderate problem	27	(41.5)	40	(44.4)	0.719
CIRS-G Psychiatric illness severe problem	19	(29.2)	12	(13.3)	**0.017**
CIRS-G Psychiatric illness extremely severe problem	0	-	2	(2.2)	….
Fatigue	54	(83.1)	84	(93.3)	0.066
Nervous^b^	22	(33.8)	27	(31.8)	0.861
Easily irritated^b^	23	(35.4)	20	(23.6)	0.145
Difficulty concentrating^b^	22	(33.8)	27	(32.1)	0.728
Anxious^b^	19	(29.2)	25	(29.4)	1.000
Feeling down^b^	33	(50.1)	40	(47.1)	0.622
Cries easily^b^	17	(26.2)	16	(18.9)	0.323
Difficulty relaxing^b^	21	(32.3)	45	(52.9)	0.862
Poor appetite^b^	22	(33.8)	52	(61.2)	**0.001**
Weight loss^b^	33	(50.8)	45	(50.6)	1.000
Loss of pleasure	38	(58.5)	30	(33.3)	**0.002**

^a^Montgomery-Åsberg Depression Rating Scale (MADRS) normal score 0–6, mild depression 7–19, moderate depression 20–34, severe depression 35–54

^b^Missing: MADRS 10 men and 10 women, nervous 5 women, easily irritated 5 women, difficulty concentrating 2 men, 6 women, anxious 1 man, 5 women, feeling down 1 man, 5 women, cries easily 5 women, difficulty relaxing 5 women, poor appetite 5 women, weight loss 1 woman, sleep disturbances 5women

^c^Cumulative Illness Rating Scale-Geriatrics (CIRS-G), (psychiatric illness also includes dementia). No problem affecting that system, Current mild problem or past significant problem, Moderate disability or morbidity and/or requires first-line therapy, Severe problem and/or constant and significant disability and/or hard-to-control chronic problems or Extremely severe problem and/or immediate treatment required and/or organ failure and/or severe functional impairment

Univariate analyses showed strong correlations between depression (MADRS ≥ 7) and dependence in ADL, poor SRH, roles (disagree being able to do the things one values) and being dissatisfied with physical and mental health (Table 3).

**Table 3:** Characteristics of hospitalized frail older people with and without symptoms of depression

**MADRS**^**a,b**^	**MADRS (≥ 7)** **(*n* = 82)**	**(%)**	**MADRS (0–6) (*n* = 53)**	**(%)**	***p*-value**^**1**^
Age: mean, (range)	85.2, (75–98)		87.1, (76–100)		0.074
Days of hospital stay, mean, (range)	12.5, (2–48)		11.5, (2–61)		0.489
Dependence in ADL	44	53.7	24	45.3	**0.031**
Poor self-rated health^b^	62	75.6	31	58.5	**0.028**
Severe problem or extremely severe problem in ≥ 3 somatic categories on CIRS-G^c^	38	46.3	22	41.5	0.581
Love and friendship (disagree can have love and friendships wanted)^b^	13	18.3	6	15.0	0.433
Roles (disagree able to do things that make one feel valued)^b^	36	54.5	10	22.7	**0.001**
Physical health (disagree satisfied)^b^	36	57.1	14	36.8	**0.050**
Mental health (disagree satisfied)^b^	18	29.0	3	8.1	**0.021**

^a^ Montgomery-Åsberg Depression Rating Scale (MADRS) normal score 0–6, mild depression 7–19, moderate depression 20–34, severe depression 35–54

^b^Missing: MADRS 20, SRH 1 depressed, ICECAP-O disagree attachment 11depressed, 13 not depressed, ICECAP-O disagree roles 16 depressed, 9 not depressed, Fugl-Meyer Life Satisfaction disagree physical health 19 depressed, 15 not depressed, Fugl-Meyer Life Satisfaction disagree mental health 20 depressed, 16 not depressed

^c^Morbidity in 3 or more organ systems (heart, vascular, hematopoietic, respiratory, eyes–ears–nose throat and larynx, upper gastrointestinal, lower gastrointestinal, liver, renal, genitourinary, musculoskeletal, neurological and endocrine) as assessed by the Cumulative Illness Rating Scale – Geriatrics. Morbidity was having severe problem and/or constant and significant disability and/or hard-to-control chronic problems or extremely severe problem and/or immediate treatment required and/or organ failure and/or severe functional impairment

^1^Mann–Whitney U/ Kruskal–Wallis tests

One variable (roles: not able to do things that make one feel valued) remained associated with depression (Table 4).

**Table 4:** Logistical Regression of factors associated with depression (MADRS^a^ ≥ 7, *n* = 82)

	**OR**	**95% CI**	**Wald**	***p*-value**
**Dependence in ADL**^b^	1.58	0.59–4.18	0.856	0.355
**Self-rated health**	1.16	0.41–3.38	0.096	0.778
**Roles (disagree able to do things that make one feel valued)**^c^	4.15	1.50–11.62	7.311	**0.007**
**Physical health (disagree satisfied)**^d^	1.16	0.40–3.41	0.077	0.781
**Mental health (disagree satisfied)**^d^	5.27	0.99–28.08	3.787	0.052

^a^(MADRS) Montgomery-Åsberg Depression Rating Scale

^b^ADL (Activities of Daily Living)

^c^ICECAP-O Capability measure for older people

^d^Fugl-Meyer Life Satisfaction Questionnaire

When comparing all participants in the intervention with the control group for mental health status at discharge the proportion on antidepressant medication was larger in the intervention group (Table 5).

**Table 5:** Indicators of mental health status in hospitalized frail older adults

	Control (*n* = 77) *n* (%)		Intervention (*n* = 78) *n* (%)		OR	CI	*p*-value
MADRS^a,b^							
Normal	26	38.2	27	40.3	0.99	0.50–1.97	0.975
Mild depression	36	54.5	34	49.3	0.81	0.41–1.59	0.540
Moderate depression	6	9.1	6	8.7	0.95	0.29–3.12	0.936
CIRS-G^c^ Psych no problem	14	18.2	11	14.1	0.74	0.31–1.75	0.491
CIRS–G Psych mild current or previous serious problem	19	24.7	11	14.1	0.50	0.22–1.14	0.099
CIRS-G Psych moderate problem	29	37.7	38	48.7	1.57	0.83–2.98	0.166
CIRS-G Psych severe/very severe	15	19.5	18	23.1	1.24	0.57–2.68	0.585
Documented depression diagnosis at discharge	1	1.3	7	8.9	7.49	0.90–62.43	0.063
Antidepressant medication	17	22.1	31	39.7	2.33	1.15–4.71	**0.019**
Documented anxiety diagnosis at discharge	0	0	2	2.6	-	-	-
Anxiety medication	18	23.4	21	26.9	1.21	0.58–2.50	0.611
Sleep medication	26	33.8	38	48.7	1.86	0.97–3.56	0.060

^a,^^b^MADRS (Montgomery-Åsberg Depression Rating Scale) normal score: 0–6, mild depression: 7–19, moderate depression: 20–34, severe depression: 35–54, MADRS missing: control 11, intervention 9

^a^CIRS-G (Cumulative Illness Rating Scale-Geriatrics)

## Discussion

Symptoms of depression were common in frail older people who were admitted to hospital for somatic care needs. Poor self-rated health, dependence in ADL, inability to do things that make one feel valued, and dissatisfaction with physical and mental health were all associated with depression, but feeling unable to do things that make one feel valued was the only factor that showed an independent association with depression. Frail older people were more than twice as likely to receive depression medication when care was provided on a CGA ward compared to a routine medical ward.

Our observed depression prevalence (61%) is nearly double that (39%) reported in a meta-analysis [5]. The discrepancy is likely related to differences in recruitment settings. Our cohort involved frail older people who required emergency medical treatment, whereas the meta-analysis was based on cross-sectional/longitudinal studies investigating depression and frailty in the general older population. Moreover, type of assessment tool and choice of cut-offs will affect prevalence figures, as will the panorama of comorbidities and degree of access to medical resources amongst study populations.

Our findings could be relevant when treating frail older people admitted to hospital for unplanned medical needs, as many may also have depressive symptoms. Older people however are often hesitant and feel uncertain about seeking help related to their mental health issues [42]. Older people with high levels and steep trajectories in frailty also tend to demonstrate increased levels and steeper trajectories in depression [43]. It is thus essential that medical staff try to detect symptoms of depression. Earlier studies [44, 45] report symptomatology may differ in men and women. In our study, men had more severe scores on CIRS-G psychiatric illness, had lower cognitive scores and were more likely to report loss of pleasure, while a greater proportion of women had poor appetite.

In an effort to understand how our participants subjectively experienced their life, the research interview included questions about well-being and life satisfaction. One question, feeling unable to do things that make one feel valued, showed an independent association with depression. Asking this one question could be fundamental in initially accessing the frail older person’s narrative relating to mental well-being. This information could aid health care staff in identifying people who might be depressed during their hospital admission, since it encompasses a person’s subjective response about their quality of life [46]. Feeling valuable relates conceptually to understanding an older person’s capability [46–48], i.e., wellbeing in terms of having the ability to ‘do’ and ‘be’ the things that are important in life.

We had anticipated that participants randomized to the CGA intervention would be more likely to receive a depression diagnosis. However, medical record review revealed that few of the frail older participants in either group had received a clinical diagnosis of depression. A recent study in an acute hospital setting in the United Kingdom (that did not examine frailty) demonstrated that many older adults with clinically relevant depression did not receive a depression diagnosis [49]. This problem is not limited to the hospital setting. In a newly published Chilean study [50] exploring non-hospitalized older adults, results showed that less than half who screened positive on a self-reported depression assessment had received a diagnosis of depression [50]. While the majority of our participants had a MADRS score indicating some level of depression, less than half were receiving medications for their symptoms of depression. In some cases, clinical depression may have been underdiagnosed. Others may have had subthreshold depression. This is a condition that commonly affects older people who have a substantial depressive symptomatology without meeting the diagnostic criteria for a depressive disorder [51]. It is important to identify and manage subthreshold depression in older patients, as this diagnosis is associated with significant difficulties in functioning, which is likely to have a negative impact on quality of life [52].

Lastly, we observed that antidepressants were prescribed twice as often in the CGA intervention group compared to the group with routine medical care. This suggests that CGA care was more holistic and comprehensive in treating frail older people’s mental health. The findings from an earlier study supported that a CGA assessment was associated with improved identification of depression among older people [21]. The assessments used in our CGA-Swed were chosen to capture a person’s medical status, functional status, social situation, their home environment and their psychological status, and included a thorough pharmaceutical review [12]. When all this is done, care is tailored to address the frail older person’s unique needs, which facilitates their independence and best possible coping skills [42]. This can potentially increase a person’s self-rated health and life satisfaction [12]. The heterogeneous nature of the frail older population must be stressed, however. Antidepressant response will vary; treatment may be beneficial for some but not for others. Even when frail older people do respond to antidepressants, symptom remission appears to have little or no impact on the degree of frailty [53]. Considering the increased risk of adverse events, antidepressants should be discontinued in older adults who do not respond [11]. Novel strategies need to be developed and tested for frail older people with depression.

A strength of this study is the focus on frail older people requiring unplanned hospital care, which is an under-researched group, especially when it comes to mental health needs. However, the fact that participants were frail and acutely ill meant that not all were able to complete all assessments and questions in the CGA-Swed RCT [12]. Hence, power was lacking for some of the analyses. Furthermore, there was no way of knowing if depression influenced the need for admission, since the study was not designed to identify this. The frail older person is not always cognizant of their wants and/or may not be confident in disclosing all their mental and social health needs when admitted to hospital [54]. Our study’s population was selected based on the need for unplanned hospital care, and results cannot be extrapolated to frail older adults in the general population. Furthermore, the study lacks data on mental health history and duration of current depressive symptoms, so that it was not possible to determine whether symptoms were short-term reactions to the stress associated with acute illness and hospital admission. The stress of getting sick and being hospitalized can be considered an adverse condition for older people and this increases the risk of depression [21]. Lastly, a weakness to this study is that people with cognitive impairments did not have a next of kin present during the assessments and data collection. If participants were unable to respond and or participate due to different frailty factors including cognition, assessments and or questions were left blank and or not completed. Future studies are warranted to better untangle the physical and mental health issues in frail older people who are acutely admitted to hospital, so health care staff can better prioritize intervention strategies.

## Conclusion

Symptoms of depression were common among frail older people with unplanned hospital admission. In our study, depressive states were underdiagnosed and undertreated to a large extent. However, people who received the CGA intervention had higher odds of receiving antidepressant treatment, which suggests that CGA improves recognition of mental health needs during unplanned hospital admissions. Further research is warranted to understand the factors involved in these gaps, and to improve depression detection and treatment in frail older people.

## Data Availability

The dataset used and analysed in the current study is available from the corresponding author on reasonable request. De-identified participant data that underlie the results reported in this article will be available to the scientific community, immediately after publication and up to five years, upon request from researchers who provide a methodologically sound proposal and attempt to achieve aims in the approved proposal. Data will be stored for 10 years at the University of Gothenburg to enable review. Proposals should be directed to the corresponding author, Theresa.westgard@neuro.gu.se. To gain access, data requesters will need to sign a data access agreement. Data is covered by the Public Access to Information and Secrecy Act and a confidentiality assessment will be performed at each individual request. Permission from the University of Gothenburg, the Institute of Neuroscience and Physiology, has to be obtained before data can be accessed.

## Abbreviations

CGA: Comprehensive Geriatric Assessment
MADRS: Montgomery Åsberg Depression Rating Scale
ICECAP-O: ICE Capability measure for older people
Fugl-Meyer Lisat: Fugl-Meyer Life Satisfaction Scale
OR: Odds Ratio
CI: Confidence Interval
ADL: Activities of Daily Living
RCT: Randomized control trial
SRH: Self-rated health
PADL: Personal Activities of Daily Living
IADL: Instrumental Activities of Daily Living
MMSE: Mini-Mental State Examination
CIRS-G: Cumulative Illness Rating Scale-Geriatrics
